# Risk of colloidal and pseudo-colloidal transport of actinides in nitrate contaminated groundwater near a radioactive waste repository after bioremediation

**DOI:** 10.1038/s41598-022-08593-3

**Published:** 2022-03-16

**Authors:** Alexey Safonov, Elena Lavrinovich, Alexander Emel’yanov, Kirill Boldyrev, Vladimir Kuryakov, Natalia Rodygina, Elena Zakharova, Alexander Novikov

**Affiliations:** 1 Frumkin Institute of Physical Chemistry and Electrochemistry, Russian Academy of Sciences, 31, Leninsky prospect, 199071 Moscow, Russia; 2 Vernadsky Institute of Geochemistry and Analytical Chemistry, Russian Academy of Sciences, Kosygin str. 19, 119991 Moscow, Russia; 3 Nuclear Safety Institute of the Russian Academy of Sciences, 52, Bolshaya Tulskaya, 115191б Moscow, Russia; 4 Oil and Gas Research Institute of the Russian Academy of Sciences, Gubkina str. 3, 119333 Moscow, Russia

**Keywords:** Biogeochemistry, Environmental sciences, Biogeochemistry, Microbial ecology, Environmental biotechnology, Nuclear waste

## Abstract

The possible role of biogeochemical processes in the transport of colloidal and pseudo-colloidal U, Np, and Pu during bioremediation of radionuclide- and nitrate-contaminated groundwater was investigated. In two laboratory experiments with water samples taken from contaminated aquifers before and post bioremediation, we found that microbial processes could cause clayed, ferruginous, and actinide colloids to coagulate. The main mechanisms are biogenic insoluble ferrous iron species formations (goethite, pyrrhotite, siderite, troilite, and ferrihydrite), the aggregation of clay particles by microbial metabolites, and the immobilization of actinides in the bacterial cells, large polymers, and iron and clayed sediments. This process decreases the risk of colloidal and pseudo-colloidal transport of actinides.

## Introduction

Subsurface uranium leaching, radiation accidents, underground nuclear explosions and improper handling of surface waste storages of radiochemical production plants and ore processing sites may cause the ingress of actinides into groundwater. The most significant problems with actinide pollution arose in the United States and Russia during the arms race in the middle of the twentieth century. Examples of such sites in the United States are the Nevada Test Site, the Yucca Mountain nuclear waste repository, the Los Alamos Laboratory, and the Hanford plant. In Russia, liquid radioactive waste (LRW) was disposed of in both deep (250–350 m) collectors^1^ and upper open repositories of solid sludges and tails^2–4^ at several radiochemical plants, including, the Siberian Chemical Combine (SCC) and the Mining Chemical Combine. Since nitrates are the main radioactive waste components in surface depositories and deep collector layers, radionuclide migration occurs at elevated salt concentrations. The presence of nitrate ions and sulfate ions promotes the oxidative environment in aquifers and a consequent migration of actinides in a dissolved oxidized form^5,6^. Moreover, carbonates also create the risk of actinides migration in highly soluble carbonate complex forms^7^. In the last 30 years, the mechanisms of colloidal and pseudo-colloidal transport of actinides under diverse geochemical conditions have been of particular interest^8,9^. Several studies indicate that colloidal transport may play a key role in the migration of uranium and plutonium as true colloidal particles and components of complex particles in ferruginous, clay, or organic complexes^10–15^. Colloids that carry radionuclides could be classified into three groups according to their origin: intrinsic colloids, primary colloids, and pseudocolloids (Buck and Bates1999). The existence of each form is primarily determined by its mineral composition, the physicochemical parameters of the groundwater, and the chemical properties of the actinide itself. In natural waters with a high content of ferruginous and clayed particles, the pseudo-colloidal form of actinide transport is likely to be the most important^16^. According to Kersting and Zavarin^17^ and Kersting, et al.^18^, the majority of the Pu at the Nevada Test Site is associated with the smallest 10–100 nm colloidal fraction, associated with Cs, silicates, and as an oxide hydrate^19^. Analysis of the groundwater from the Los Alamos National Laboratory discharged since the 1940s showed that 85% of Pu was bound to particles of the size of 25–450 nm, and 31% of Am was bound to colloid particles the size of which exceeded 5 nm^16^. At Yucca Mountain, strongly sorbed radionuclides such as americium and thorium are most affected by pseudocolloid formation and transport. In contrast, less strongly sorbed radioelements, such as uranium and neptunium, are not affected significantly by colloid transport. An essential role of iron as a product of steel corrosion in the formation of uranium and plutonium colloidal particles is noted in work on the assessment of colloidal formation during the storage of uranium fuel.

Penetration of nitrates and other components into the surface and submerged water-bearing aquifers may enhance the microbial processes^20,21^. At some plants, the microbial community was stimulated with organic substrates to immobilize uranium and other radionuclides and remove nitrates^22–24^. Multiple studies on bioremediation of uranium-containing waters commonly describe the “biogenic uraninite” form of uranium IV as a poorly soluble X-ray amorphous phase^25,26^. The formation of several biogenic phosphate phases is also noted^27^. However, no data on similar forms of plutonium and neptunium could be found. The role of biogeochemical processes in colloidal and pseudo-colloidal actinide transport remains insufficiently studied. Biogenic processes stimulated by nitrate reduction, for example, might have stabilized pseudo-colloidal actinide particles due to production of exopolysaccharide metabolites^28,29^ or biogenic nano colloidal uraninite^30^. The formation of biogenic ferruginous particles^31^ may result in new actinide-transporting colloidal phases.

An aquifer polluted with nitrates and actinides near the mothballed SCC RW repository was taken into consideration in the present study. During 2015, bioremediation of the named aquifer was conducted. Bioremediation involved a single stimulation of the microbial community with a mixture of acetate and whey. This led to a significant decrease in the redox potential of the system and a temporary reduction of nitrate ion concentrations to values below the maximum allowable concentrations for in-place conditions^32^. Decreased uranium content was observed in samples taken after the bioremediation. Such results have shown promising possibilities for developing a biogeochemical barrier for radionuclides under the conditions of the aquifer in consideration. These possibilities assume the continuous stimulation of the microbial community and formation of local areas with reducing conditions so that elements are immobilized in poorly soluble form.

The migration of actinides in groundwater after bioremediation requires the development of the multifactor and multi-parametric models, which shall assume the consequences of the actinide’s mineral forms, oxidation states, and species in water, including colloid and pseudocolloid forms. The goal of the present work was to assess the role of metabolic products in water samples taken from an aquifer where in-situ bioremediation was previously conducted. The role of named products is to be assessed in experiments that model the bioremediation process under conditions of stability of colloidal and pseudo-colloidal ferruginous and clayed phases containing plutonium, neptunium, and uranium.

## Materials and methods

Water samples were taken from an aquifer located near the suspended surface RW repository (Siberia, Russia) by means of an observation well (12 m depth) after pumping one and a half well volumes. Sample 1 was taken at the beginning of 2015 before the in situ bioremediation, sample 2 in the middle of 2016, and sample 3 in 2017, two years after the bioremediation process was conducted. The results of measuring the main physical and chemical parameters of water are given in Table 1. The laboratory experiment was carried out in 2018. The samples were hermetically sealed and stored at a temperature of + 4 °C in a refrigerator. The values of pH, Eh, and conductometry were determined at the time of sampling. After each sampling, sequential filtration of samples was carried out using syringe filter attachments and cellulose acetate membranes with pore sizes of 2.4, 1.2, 0.45, 0.22, 0.1, and 0.05 μm.

**Table 1:** Parameters of the groundwater samples, mg/l.

Sample	1	2	3
pH	6.58	6.2	7.0
Eh	65	− 175	70
Permanganate oxidizability, mg O_2_/L	13.10	68.9	8.94
TOC	11.9	57.5	7.69
Salinity	3952.0	1567	1970.0
Fe (total)	0.25	2.9	1.3
Na^+^	604.0	570	257.0
K^+^	3.09	11.1	9.8
Ca^2+^	316.60	19	109.2
Mg^2+^	63.20	45	72.5
NH_4_^+^	< 0.05	15.9	7.64
NO_3_^−^	2517.0	320.7	970.0
SO_4_^2−^	172.40	23.6	25.1
Fe (total)	2.89	7.91	3.12
Cl^-^	4.52	5.1	5.37
HCO_3_^−^	231.0	987.5	372.2
NO_2_^−^	< 0.2	15.6	5.2
Suspended matter content	12	2,8	3,6
U	1.1	0.08	0.14
Pu Bq/L	0.7	*	*
∑α- activity Bq/L	12.59	0.9	0.55
∑β- activity, Bq/L	28.7	1.1	8.2

The concentrations of cations and anions were measured on a Capel-205 (Russia) new generation capillary electrophoresis system, U by ISP-MS, Pu, ∑α- and ∑β- activity by radiometry.

*Below detection level.

Laboratory simulation tests (table SI-1 supplementary file).

In the first experiment, molecular hydrogen added to the gas phase was used as an electron donor. Water samples 1 and 3 were used as the liquid phase. The experiment was carried out for 30 days in a hermetically sealed 50 ml vial at a temperature of 20 °C.

For the second laboratory simulation, water sample 1 (natural water NW) and model water (MW) of the composition mg/l were used: NaHCO_3_—25.2; MgSO_4_ * 7H_2_O—36.6; CaCl_2_ * 6H_2_O—233.8; and MgCO_3_—3.2 in which 10% of the volume was added to sample 1 of natural water to introduce a source of microflora. 1 g/L of sodium acetate and 1 g/l of glucose (MWO and NWO) was added to some of the MW and NW samples for bacteria stimulation. In a series of model samples, 100 mg/L of bentonite clay (MWCl) 10th Khutor deposit, Khakassia)^33^ and 50 mg/L FeCl_3_ (MWI) were added. Argon was used as the gas phase in the headspace.

Actinides (^233^U, ^237^Np, and ^239^Pu) were added in all samples in the concentrations of 10^–8^ mol/L per sample before the laboratory experiment began.

The laboratory experiment took place for a month at a temperature of 20 °C in hermetically sealed penicillin vials. In all cases, the volume of the liquid phase was 50 ml.

### Analytical techniques

The concentrations of cations and anions were measured on a Capel-205 (Russia) new generation capillary electrophoresis system. Identification and quantitation of the analyzed cations and anions were performed by indirect detection by measuring UV absorption at 254 nm. Electrophoresis was performed in untreated fused-silica capillaries of 60-cm length (effective length, 50 cm) and 75 μm internal diameter. The capillary was held at 20 °C and the applied voltage was + 13 kV for cations or –17 kV for anions.

Concentrations of microelements (including U) in the solution were determined by mass spectrometry with inductively bound plasma on Element-2 (Thermo Scientific, USA). The microelements measurement was carried out in samples acidified with nitric acid (up to pH = 1).

The determination of the total alpha and beta activity of natural waters was carried out by measuring the integral count of alpha and beta particles from counting samples prepared from water samples after a preliminary procedure for their concentration. Concentration was carried out by evaporation of 1.0 dm^3^ of an aqueous sample to a dry residue, sulfation of the dry residue of the sample with sulfuric acid and subsequent calcination in a muffle furnace at 1250 °C to remove residual sulfuric acid. The calcined precipitates were used to prepare a counting sample, which was measured on a Berthold LB2046 low background alpha, beta radiometer. The alpha and beta radiometers were calibrated using bulk reference sources with certified specific activity of alpha and beta emitting radionuclides.

The determination of the 239Pu and 233U in natural water was carried out by alpha-spectrometry, (Alphar-analyst) (Canberra, Australia). Actinides were isolated from a 1 dm^3^ sample preliminarily acidified with nitric acid by their co-precipitation with iron hydroxide. The 236Pu and 232U labels were preliminarily added to the solutions. Radiochemical separation of radionuclides was carried out by extraction chromatography. The column material was silica gel with octylphenyl-N,N-diisobutylcarbamoylphosphine oxide (SMPO). The sorbed actinides were sequentially eluted with solutions of various compositions: plutonium (III)—with a hydroquinone solution in hydrochloric acid, uranium (VI)—with an ammonium oxalate solution. After decomposition of organic substances with concentrated nitric acid, a counting sample was prepared by electrodeposition on a steel substrate (layer thickness no more than 0.01 μm). The lower limit for measuring the specific activity of controlled materials is 0.1 Bq in a sample.

Concentration of ^233^U in laboratory experiments was determined by liquid scintillation technique (Tri-Carb-3180 TR/SL liquid scintillation spectrometer) ("Perkin-Elmer," USA). For ^239^Pu determination alpha-spectrometry (Alphar-analyst) (Canberra, Australia) was used. ^237^Np concentration was determined by the luminescent method LFF-5 (luminescent filter photometer) produced by GEOKHI RAS^34^. The procedure of direct luminescence determination of actinides consisted in the application of an aliquot portion of analyzed solution onto the crystal phosphor (the volume of the analyzed sample varies from 0.02 to 0.1 mL), which was put into a quartz beaker, with subsequent drying under an IR lamp, annealing on an electric furnace for 45 min and final annealing in a muffle furnace for 1 h at 900 °C. Then, the intensity of sample luminescence for 237Np(V) was measured on an LFF-5 analyzer within a short distance IR range (1713 nm). The most efficient crystal phosphor for 237Np(V) was PbMoO4 (Myasoedov and Novikov, 1997) (detection limit 0.3 pg).

Carbohydrate (polysaccharides) determination was carried out by the phenol–sulfuric acid method according to Dubois^35^.

Protein content was measured with the Folin phenol reagent according to Lowry^36^.

Eh, and pH were determined with an Anion ion meter using the relevant electrodes (Econix Expert, Russia).

Organic matter in the liquid was measured using an Elementar Vario EL III CHN analyzer (Elementar Analysensysteme GmbH, Germany). The content of organic matter was measured in the unfiltered sample and in each filtrate with successive filtration.

The content of suspended solids in the samples was determined by the standard method of gravimetric filters after filtration of 100 ml of water.

The size of the cells, colloidal particles, and zeta potential were determined by the dynamic light scattering method using the Compact-Z particle size and zeta potential analyzer (Photocor, Russia). This method is based on measuring the temporal fluctuations in the scattered light intensity. The device operates within the ISO13321 standard, which indicates a measurement accuracy of at least 2%.

Light scattering intensity was determined by the Zetasizer Nano ZS, Malvern Panalytical.

The size of colloidal particles in the environmental samples and model experiments was determined by step-by-step filtration with syringe-mounted Vladipor (Russia) filters 2.4, 1.2, 0.8, 0.4, 0.22, 0.1, and 0.05 µm in diameter.

Electron micrographs were obtained by the JEOL JSM-T330A scanning electron microscope (Japan) with acceleration voltage 25 kV and the Tracor Northern Z-Max 30 Series TN-5502 N Energy-Dispersive Spectrometer.

The speciation of actinides in water samples was assessed by thermodynamic modelling in the PhreeqC 2.1software with the *llnl.dat* thermodynamic database^37^. The saturation indices (SI) were determined as follows: SI = logIAP—logK_s_, where IAP is a product of activities of the relevant ions and K_s_ is the equilibrium constant. At SI > 0 formation of the studied phase is predicted. The amount of each actinide element added per sample was 500 µg.

The microbiological parameters of the collected samples were presented in previous articles^32,38^. The presence of the following bacteria in aquifer samples was revealed: aerobic organotrophic, anaerobic fermenting, iron-reducing, and denitrifying bacteria of the phyla *Proteobacteria* (genera *Acidovorax*, *Simplicispira*, *Thermomonas*, *Thiobacillus*, *Pseudomonas*, *Brevundimonas*, and uncultured *Oxalobacteraceae*), Firmicutes (genera *Bacillus* and *Paenibacillus*), and Actinobacteria (*Candidatus Planktophila*, *Gaiella*).

### Declarations

This research does not contain any studies with human participants or animals performed by any of the authors. Authors can confirm that all relevant data are included in the article and/or its supplementary information files.

## Results and discussion

### Characteristics of environmental samples before and after bioremediation

**Figure 1:**
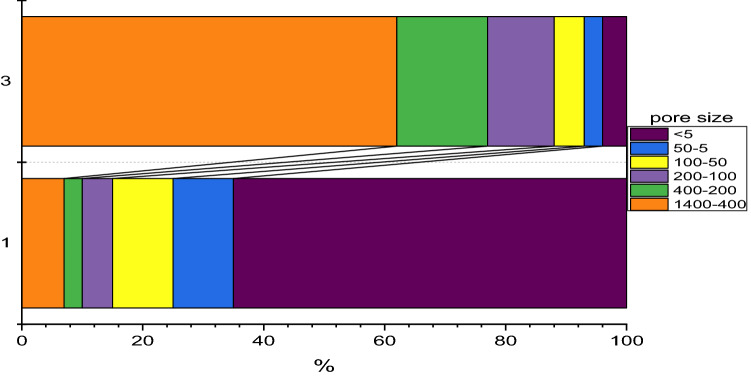
Percentage distribution of uranium in the filtrate during sequential filtration of samples 1 and 3. Concentrations of U in the filtrates were determined by the ICP-MS method.

Table 1 lists the parameters of the samples collected from the upper aquifer (12 m) at three-time points. In sample 1, before bioremediation, the content of nitrate ions reached 2517 mg/L. Against this background, in an oxidizing environment, a high content of uranium up to 1.1 mg/L and plutonium up to 0.7 Bq/L was observed. The content of organic matter did not exceed 5.9 mg/L. The suspension contained a significant amount of clay particles. Uranium in sample 1 was predominantly in dissolved form or nanoaggregates less than 5 nm in size (Fig. 1).

In sample 2, a year after the injection of organic matter, the content of nitrate ions reached 320 mg/L, while the values of the redox potential continued to remain in the reduction region (− 175 mV) as they were 3 months after bioremediation. The content of organic matter reached 57.5 mg/L. The uranium content dropped to 80 μg/L, and the plutonium content was below the detection limit of the device.

2 years after injection (sample 3), the content of nitrate ions increased to 970 mg/L, the redox potential entered the oxidizing region and reached + 70 mV, while no significant release of uranium into solution occurred. According to the distribution scheme of uranium (Fig. 1), most of it was associated with large particles of more than 400 microns in size of clay and ferruginous nature. The plutonium content was below the sensitivity of the method. Thus, despite the fact that after a single injection of organic matter, after two years the content of nitrate ions increased markedly and the value of the redox potential returned to the oxidizing region. Nevertheless, it should be mentioned no significant remobilization of uranium and plutonium occurred. It is important to note that according to the data in Table 1, a decrease in the content of suspended matter was observed in the course of bioremediation. A discussion of the content of organic matter in the suspended matter will be carried out in the next section.

**Figure 2:**
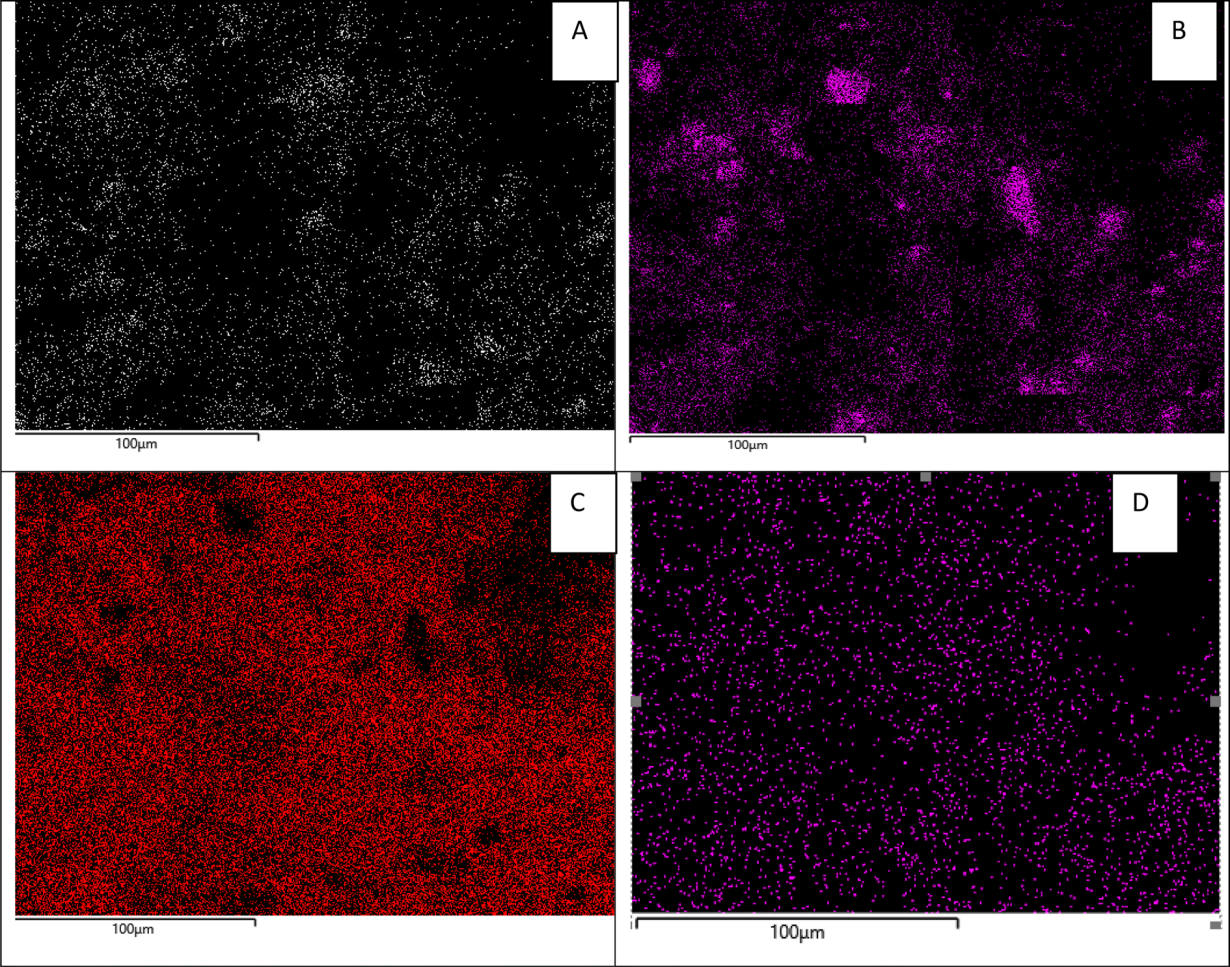
Electron micrographs of the filters with a pore size of 2400 nm surface after sample 3 filtration with elements maps **(A)** Al, **(B)** Si, **(C)** Fe, **(D)** U (SEM EDX analysis).

Figure 2 shows electronic maps of micrographs of a filter with a maximum pore size after filtration of sample 3. It has been established that U is mainly associated with large particles (suspensions) of aluminosilicate and ferrous nature. The distribution of Al, Si, Fe and U on the surface of the filter cake was fairly uniform.

Although at low plutonium concentration it was not possible to see it by the SEM EDX method on clays, it is well known that clay minerals montmorillonite and kaolinite could have been carrier phases for Pu^39^. In work on the analysis of colloidal transport of radionuclides in groundwater at Yucca mountain^40^ uranium was found to be dominantly associated with an unidentified phase rich in Si and Fe while Pu was shown to be preferentially adsorbed onto Mn-oxides in the presence of Fe-oxides.

### Laboratory simulation of biogenic associative colloids formation in environmental water samples, stimulated by H_2_

In a laboratory experiment with environmental samples, molecular hydrogen was used to stimulate microbial processes in order to avoid changing the content of the organic matter.

**Figure 3:**
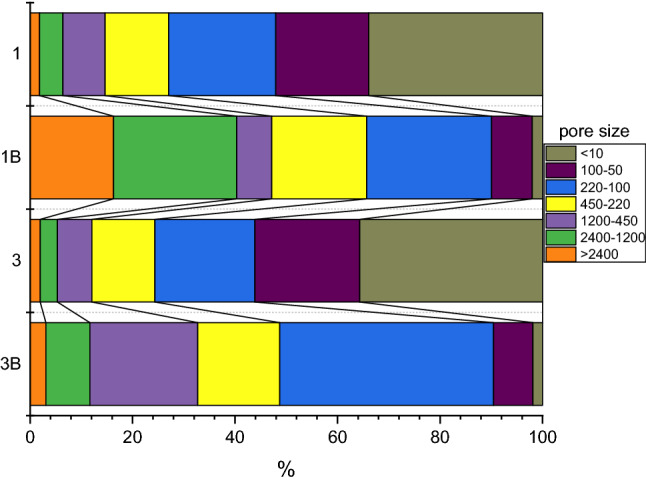
Organic matter distribution by particle size (nm) in samples 1 and 3 before and after (B) microbial activation. Organic matter in the filtrate after each filtration step was measured using an Elementar Vario EL III CHN analyzer.

Filtration studies (step-by-step filtration, Fig. 3) revealed that only 8% of organic matter in sample 1 was represented by suspended particles over 1200 nm in size. These were bacterial cells and other large particles (fulvic and humate acids, etc.). More than 50% of organic matter was in soluble form or in the form of colloidal particles up to 100 nm. In general, the distribution of organic matter in sample 3 was similar to sample 1—about 60% of organic matter was in dissolved or colloidal form and about 10% in the form of large particles.

An organic carbon content of 100 and 200 mg/L was observed in samples 1 and 2, respectively, after microbial activation by molecular hydrogen.

After day 30 of incubation in sample 1 and after microbial processes, there was a noticeable increase in the content of large organic particles; their contribution reached 50%. In this case, the content of dissolved organic matter and organic particles of colloidal size decreased noticeably (their total contribution did not exceed 10% probably due to their consumption or aggregation into larger fractions). The content of organic particles with a size range of 220–450 nm had noticeably increased.

In sample 3, a noticeable decrease in dissolved and colloidal organic matter was also noted; the content of organic particles of 220–100 nm and particles of 1200–400 nm increased markedly. We believe that the increase in organic particles in both samples in the range of 100–1200 nm is associated with an increase in the content of bacterial cells. Changes in the intensity of light scattering provided the most relevant information (Table 2).

**Table 2:** The intensity of light scattering (kHz) by suspended particles of different fractions before and after day 30 of the ongoing microbial process in the stratal water (Light scattering intensity was determined by Zetasizer Nano ZS, Malvern Panalytical).

Particle fraction size, nm	1 before	1 after	2 before	2 after
< 2400	30 ± 10	300 ± 90	40 ± 10	100 ± 30
2400–1200	30 ± 10	50 ± 10	40 ± 10	140 ± 40
1200–450	30 ± 10	50 ± 10	50 ± 10	200 ± 60
450–220	40 ± 10	40 ± 10	30 ± 10	200 ± 60
220–100	20 ± 10	40 ± 10	30 ± 10	150 ± 40
100–50	20 ± 10	20 ± 10	10 ± 5	140 ± 40
> 10	~ 0	~ 0	~ 0	~ 0

In sample 1, before stimulation, the intensity of light scattering was at its maximum in the filtrate at 450–220 nm. In the filtrate less than 10 nm, light scattering was not detected. In filtrates larger than 450 nm and 220–50 nm, the values of the light scattering intensity were close. After microbial activation with hydrogen, a tenfold change in the intensity of light scattering was observed in the filtrate with particles larger than 2400 nm. Also, there was an almost twofold increase in filtrates with a particle size of 450–2400 nm, which is probably associated with the appearance of cells in the solution.

In sample 2, before microbial activation, the maximum intensity of light scattering was observed in the filtrate with particle sizes in the range of 450–1200 nm. After microbial activation, the intensity of light scattering significantly increased in all filtrates. It is important to note that the light scattering of particles with a size characteristic of colloids (50–100 nm) increased by more than 10 times. The different behavior after hydrogen activation of two samples can probably be explained by the fact that in sample 3 the microbial community was initially more active after the injection of organic matter into the formation. In both samples, a noticeable increase in the content of coarse suspensions may indicate the agglomeration of clay suspensions by microbial polysaccharides. According to Ivanov et al.^41^, a similar process is observed for soil and clay particles.

### Laboratory simulation of the formation of biogenic associative colloids in model and environmental water samples with actinides

The second series of experiments was carried out to evaluate the behavior of U, Np, and Pu upon activation of microbial processes. At the first stage of the laboratory simulation, a significant enlargement of large particles possibly caused by the agglomeration of natural clay and ferruginous particles due to microbial polysaccharides in natural samples was found. An important task of the second stage of the work was to assess the contribution of ferruginous and clay particles to the distribution of actinides over particles with different sizes in model solutions.

When activating the microbial community in groundwater, a mixture of whey and acetate was used. However, in a laboratory simulation of this process, we decided not to use such a complex multicomponent substrate like whey. The whey contained a lot of organic suspensions and its use in this experiment would have led to even more uncertainties. A mixture of highly soluble sodium acetate and glucose substrates was added to the samples.

Table 3 shows the data on the content of polysaccharides and proteins in solutions during microbial processes in samples.

**Table 3:** Polysaccharide (A) (mg/L) and protein (B) (mg/ml) concentrations in the model solutions during incubation. Polysaccharide determination was carried out by the phenol–sulfuric acid method according to Dubois ^34^. Protein content was measured with the Folin phenol reagent according to Lowry ^35^.

Sample	Incubation time, days												
	0		5		10		15			20		30	
	A	B	A	B	A	B	A	B		A	B	A	B
MWO	0.1	*	0.2	0.2	0.13	2.4	0.27	1.9		0.25	1.2	0.19	0.4
MWClO	0.11	*	0.3	1.3	0.15	3.0	0.29	2.5		0.21	1.2	0.13	0.4
MWIO	0.1	*	0.3	1.5	0.17	2.5	0.33	2.2		0.29	1.5	0.16	0.7
NWO	0.13	0.12	0.5	1.4	0.18	3.5	0.34	2.1		0.28	1.7	0.22	0.9

*Below range 0.05 mg/l.

No significant increase of cells or polysaccharide content was recorded in samples with no organic matter additions. A low protein content was found in the sample NWO, which indicates that some content of cells remained in it after bioremediation. An increase in the concentration of the biomass, with peak values on day 10 and polysaccharides on day 15, was observed in all samples with additions of organic matter (O) (Table 3). The maximum accumulation of polysaccharides and protein was observed for the natural sample.

On the 30th day of the experiment, there was no visible sediment in the MW sample, in the rest of the samples, there was a large amount of sediment at the bottom of the test tubes. At the same time, the solution looked almost transparent in both the MW model water sample and the MWIO sample with added iron. The average hydrodynamic radii of colloidal particles were obtained on days 3, 7, 14, 21, and 28 of the experiment (Table 4). In model water samples without added organic compounds, colloidal particles were not formed. However, by the end of the experiment, particle formation was observed. This was probably due to the transformation of colloidal matter originating from the natural water aliquot or as a result of low microbial activity.

**Table 4:** Hydrodynamic radii of colloidal particles during the experiment, nm (The measurement accuracy was at least 2%.).

Sample	Incubation time, days				
	5	10	15	20	30
MW	–	–	–	–	20
MWO	90	120	70, 150*	40, 170*	110
MWCl	130	80	90	100	160
MWClO	130, 25*	130	100	100	110
MWI	130	130	100	100	110
MWIO	100	150	160	90	–
NW	75	100	120	120	140
NWO	50	75	90	170	400

We employed the Dynamic light scattering method using the Compact-Z particle size and zeta potential analyzer (Photocor, Russia).

*Two groups of particles formed.

In the presence of glucose and acetate, the emergence of the colloidal phase and a gradual increase in particle size were observed from the fifth day of incubation. The average stable hydrodynamic radii of the particles amounted to ~ 100 nm. In the presence of clay, stable colloids with the average hydrodynamic radii of 80–90 nm were formed. Stimulation of microbial processes with glucose and acetate resulted in increased particle size and partial sedimentation (samples MWO through day 20, MWIO through day 15, and NWO through day 30). After that, the sedimentation of large particles took place, and particles of smaller sizes remained in the solution.

The addition of iron to the model system resulted in the formation of the particles with hydrodynamic radii of ~ 100 nm. The stimulation of the biological processes resulted in increased particle size, the formation of new particles (by day 21), and complete particle sedimentation by day 30.

An important parameter used to evaluate the stability of colloidal particles in the system is the value of particles’ zeta potential. When no organic matter was added, the charge of preliminarily filtered 100–50 nm particles equaled − 29, − 26.2 mV in model water, and − 16, − 12 mV in natural water, which indicates low stability of such particles (see Table 2 Supplementary). A shift in charge of particles towards zero and positive values was observed when microbial processes were running, and this hints at the stabilization of particles in the solution.

**Figure 4:**
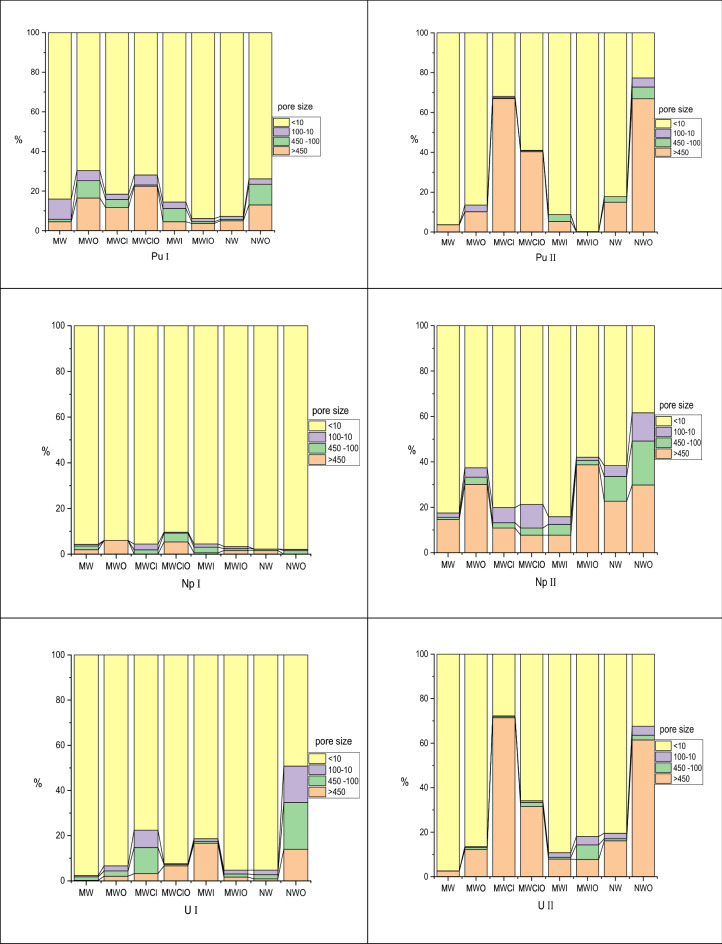
Actinide distribution by size of colloidal particles in solutions of different nature depending on the incubation time, normalized % in the filtrate. (I-before, II-after microbial stimulation on day 30). Actinides (233U, 237Np, and 239Pu) were added in the concentrations of 10^–8^ M/l per sample. Concentrations of ^233^U ^239^Pu were determined by liquid scintillation (Tri-Carb-3180 TR/SL liquid scintillation spectrometer) ("Perkin-Elmer," USA).

The diagrams of actinide distribution by size of colloidal particles in solutions of different nature before and after microbial stimulation on day 30 are shown in Fig. 4.

In the model water Pu(IV) forms true colloidal associates (up to 50%) due to deep hydrolytic polymerization. Np(V) was also partially sorbed due to slight disproportionation (by 10%). U(VI) was a stable component of soluble carbonate complexes. In the model water, increased pH and decreased Eh result in the occurrence of 99% Pu, 30% Np, and 10% U within large colloidal particles. Ultrafiltration, however, is not suitable for the assessment of the possible actinide reduction and biosorption contribution to the process of colloid formation.

The microbiota and clay promote the stabilization of Pu, U, and Np in large colloidal particles. The addition of iron had no effect on actinide colloid formation, although iron caused a significant increase in neptunium colloid formation in the presence of the microbiota. This is probably due to the formation of iron-polysaccharide complexes^42^, which also have a high ability to chelate actinides.

In Bentley^43^, high-efficiency Pu(V) adsorption onto colloids about 100 nm in size–including hematite, silica, and montmorillonite in natural and synthetic Yucca Mountain water–was mentioned. Pu sorption onto iron oxides such as hematite is strong and irreversible, but only 50% of the Pu(V) was sorbed on the montmorillonite. The time dependence of the sorption onto the clay suggests a more complicated interaction than occurs with oxide minerals. They also discussed the desorption of Pu(IV) and Pu(V) from various colloids: Pu(IV) exhibits a greater tendency to desorb than Pu(V).

According to Silva and Nitsche^44^, conditions for Np(V) (intrinsic) colloid formation in environmental waters would usually not be achieved, and Np pseudocolloid transport may be more important. In this case, Np (V and IV) could be sorbed onto environmental colloids such as Fe(OH)_3_ SiO_2,_ humic and clay particles^45^.

Thus, microbial processes may result in the coagulation of natural colloids due to the development of a weak negative surface charge. Tinnacher, et al.^39^ also mentioned that organic matter can modify the surface charge and characteristics of particle and colloid aggregates, depending on their size. For example, large surface-active organic molecules such as polysaccharides act to bind colloid particles together and thus cause colloid instability. In addition, microbial polymers and exopolymers themselves can not only lead to adhesion of colloids and suspended particles, but also increase the sorption of actinides on them due to various organic functional groups^38,46–48^.

After termination of the biogenic processes, the formation of large uranium-, plutonium-, and neptunium-containing particles associated with cells^49^ and large biopolymers (protein-polysaccharide biofilms)^50,51^ was observed. This results in decreased migration activity of the actinides since it is known that the transport of colloids is strongly influenced by their size and water filtration parameters. With an increase in the size and charge of a colloidal particle, the risk of its migration is significantly reduced^52^.

The stability of colloidal solutions depends on many factors: the size and concentration of particles of a substance, temperature, and the presence of electrolytes. An increase in the content of two and three charged ions in systems can lead to rapid coagulation of colloidal particles. The addition of bivalent cations (e.g., Ca^2+^, Mg^2+^) during bioremediation should potentiate this process and may become an efficient mechanism for decreasing the risk of active migration of radionuclide-associated particles.

### Thermodynamical modelling of the species of radionuclide occurrence in the course of biotransformation

It is well known that the nucleation process of biogenic iron oxyhydroxides (leading to their mineralization) is closely related to the organic matter of exopolysaccharides of biofilms. Through strong mineral binding (high *f*_eq_), microbial polymers can decrease the nucleation barriers for ferrihydrite and direct nucleation on the polymers^53^. The mineralization process in microbial exopolysaccharide sediments of iron and associated actinides^54^ can serve as a reliable anti-migration biogeochemical barrier, an important consequence of bioremediation.

To assess the possible contribution of biomineralization processes to the increase in the size of suspended particles which we observed in experiments, as well as to theoretically determine the forms of actinides during the development of the microbiota, thermodynamic modelling was carried out. The data on pH and Eh changes during the process of colloid formation and actinide incorporation into associative particles (and possibly into true colloidal particles as well) are listed in Table 3 (supplementary).

The most notable pH and Eh changes occurred in the presence of the microbiota, which was probably due to an increase in the number of bacteria. The pH increased moderately, while Eh values changed to negative, potentially creating the conditions for a shift of actinides’ oxidation states to lower ones. Since Ac(IV) is the most sorbed form of actinides, this may promote their association with colloidal materials of various natures^55^.

Speciation of elements, including dissolved species and the phase saturation indices, was calculated for 500 µg/L U, Pu, and Np in the Sample NWO (natural sample 2) (Table 5). The species of actinides and iron after microbial processes were calculated with an account for the following parameter changes: pH increase by 1, Eh decrease by 100 mV, complete denitrification, and sulfate reduction.

**Table 5:** The major species of actinides and iron in the liquid (M) and solid phases (Si saturation indexes) in the aquifer, after microbial treatment.

	Sample 2, before			
	U	Np	Pu	Fe
**Dissolved species, M**	U(OH)_4_ 1.6 × 10^–8^ UO_2_^+^ 1.07 × 10^–10^ **UO**_**2**_**(CO**_**3**_**)**_**2**_^**–2**^ 1.13 × 10^–6^ **UO**_**2**_**(CO**_**3**_**)**_**3**_^**–4**^ 7.8 × 10^–7^ UO_2_CO_3_ 9.4 × 10^–8^ UO_2_(OH)_2_ 3.8 × 10^–8^ (UO_2_)_2_CO_3_(OH)_3_^-^ 1.4 × 10^–8^	**Np(OH)**_**4**_ 1.9 × 10^–6^ Np(OH)_3_^+^ 2.7 × 10^–9^ **NpO**_**2**_^**+**^ 2.0 × 10^–7^ NpO_2_CO_3_^-^ 9.8 × 10^–10^ NpO_2_OH 3.5 × 10^–10^	**PuSO**_**4**_^**+**^ 2.09 × 10^–7^ Pu(SO_4_)_2_^-^ 4.2 × 10^–8^ PuOH^+2^ 8.1 × 10^–9^ **Pu(OH)**_**4**_ 1.3 × 10^–7^	**FeHCO**_**3**_^+^ 2.1 × 10^–6^ FeSO_4_ 4.1 × 10^–8^ FeCO_3_ 3.1 × 10^–8^ FeOH^+^ 1.5 × 10^–9^
**Phase, SI**	UO_2.25_(beta) 1.39 UO_2.3333_(beta) 2.36 Uraninite UO_2_ 1.57	Np(OH)_4_ 2.63 NpO_2_ 11.48	Pu(OH)_4_ 2.19 PuO_2_ 10.48	Goethite 0.59 Hematite 2.11

	Sample 2, after			
	Solution			
	Fe	Np	U	Pu
Dissolved species, M	**FeHCO**_3_^+^ 1.9 × 10^–6^ **FeCO**_3_ 1.6 × 10^–6^ FeOH^+^ 3.6 × 10^–8^ Fe(OH)_3_ 3.9 × 10^–9^ Fe(OH)_4_^−^ 2.4 × 10^–10^	**Np(OH)**_4_ 2.1 × 10^–6^ Np(CO_3_)_5_^–6^ 1.8 × 10^–10^ NpO_2_^+^ 2.4 × 10^–10^ NpO_2_CO_3_^-^ 1.4 × 10^–10^	U(OH)_4_ 3.0 × 10^–8^ **UO**_2_**(CO**_3_)_3_^–4^ 2.0 × 10^–6^ UO_2_(CO_3_)_2_^–2^ 3.4 × 10^–8^	PuOH^+2^ 6.0 × 10^–10^ **Pu(OH)**_4_ 2.1 × 10^–6^ Pu(OH)_3_^+^ 2.2 × 10^–10^

	Phases			
Phase, SI	Phase	SI	Np(OH)_4_ 2.67	UO_2.25_ 1.14
	Calcite 1.66	Huntite 1.73	NpO_2_ 11.53	UO_2.25(beta)_ 1.06
	Aragonite 1.52	Magnesite 0.58	Pu(OH)_4_ 3.38	UO_2.3333(beta)_ 1.30
	Dolomite 3.97	Monohydrocalcite 0.86	PuO_2_ 11.67	Uraninite 1.84

Significant values are in bold.

In accordance with previous thermodynamical modelling experiments after microbial processes, ferrous iron in hydroxide, sulphide, and carbonate forms were formed, and precipitation of goethite, pyrrhotite, siderite, troilite, and ferrihydrite mineral phases occurred^56–59^. These new sorption phases could cause additional actinide removal from solutions^60–64^. Thus, one of the reasons for a significant increase in the size of actinide-containing particles in model samples with the addition of iron and organic matter, as well as in samples of natural water, may be the formation of ferruginous minerals.

Prior to microbial treatment, U was expected to be present as di- and tricarbonate complexes. Np occurred as a neptunoyl ion or as a relatively poorly soluble hydroxo complex. Plutonium was expected to occur as sulfate and as a hydroxo complex. Microbial processes resulted in uranium remaining as a tricarbonate complex ore as a poorly soluble hydroxide. Plutonium and neptunium were present in all aquifers as oxyhydroxides, which can be attached to mineral surfaces and various hydroxyl phases^65,66^.

It is important to note that although thermodynamic modelling predicted the formation of soluble carbonate phases of uranium and neptunium after microbial processes, in fact, no significant increase in the contribution of soluble fractions was observed in the laboratory experiment. It should be added that the solution contains supersaturated calcium and magnesium carbonate phases (calcite, aragonite, dolomite), which can also be sorption phases for most actinides^67^. In addition, it is known that carbonate phases such as calcite are actively formed in biofilm exopolysaccharides, contributing to their biomineralization^68^. Thus, the formation of carbonate polysaccharide aggregations in the solution can also contribute to their sedimentation together with actinides.

## Conclusions

As a result of bioremediation of underground waters with nitrate contamination in the area of the radioactive waste storage in situ, it was possible to significantly reduce the concentration of dissolved uranium (more than 10 times) and plutonium (to values below the detection limits of the device). Analysis of samples taken a year later and 2 years after the bioremediation process made it possible to establish that despite the increase in nitrate concentrations and the transition of the redox potential to the reduction region, significant dissolution of uranium and plutonium did not occur. Before bioremediation Uranium was predominantly in dissolved form or nanoaggregates less than 5 nm in size. According to the distribution scheme of uranium (Fig. 1), most of it was associated with large clay and ferruginous particles of more than 400 microns in size.

It is important to note that microbial processes resulted in the more extensive formation of poorly soluble phases like uraninite (UO_2_, NpO_2_, PuO_2_). Microbial processes led to the occurrence of ferrous iron carbonates and hydrocarbonates, and to the precipitation of goethite, pyrrhotite, siderite, troilite, and ferrihydrite mineral phases. Microbial processes were able to cause coagulation of environmental colloids due to the formation of a weakly negative surface charge. The addition of bivalent cations (e.g., Ca^2+^, Mg^2+^) during bioremediation should potentiate this process and may become an efficient mechanism for decreasing the risk of active migration of radionuclide-associated particles^69,70^.

After the termination of biogenic processes, large particles containing uranium, neptunium, and plutonium associated with the cells and cells’ metabolites (protein-polysaccharide, etc.) were observed. The occurrence of such particles results in decreased migration activity of the metals. Aggregation of actinide complexes with organic and inorganic colloidal particles in the course of microbial activation results in increased particle size and may hinder their migration in the groundwater-bearing aquifers.

## Supplementary Information

Supplementary Information.
