# Supplementary material for: Risk of colloidal and pseudo-colloidal transport of actinides in nitrate contaminated groundwater near a radioactive waste repository after bioremediation
Source: Sci Rep. 2022 Mar 16;12:4557. doi: 10.1038/s41598-022-08593-3 (PMC8927300; doi:10.1038/s41598-022-08593-3)
Supplement: Supplementary file 1 — Supplementary Information. [file 41598_2022_8593_MOESM1_ESM.docx]

| Samples, medium | additives | Electron donor | Gas phase, | Incubation time, days | treatment | Measurements |  |
| --- | --- | --- | --- | --- | --- | --- | --- |
| Natural water samples experiments | | | | | | | |
| NW 1 and 3 | no | H_2_ | H_2_ | 30 | step-by-step filtration with syringe- filters 2.4, 1.2, 0.8, 0.4, 0.22, 0.1, and 0.05 µm –in diameter | Elements in filtrate solution (including U): ICP-MS  Polysaccharides: phenol–sulfuric acid method  Protein: Folin phenol reagent according to Lowry  Total organic carbon: CHN analyzer |  |
| Model and natural water experiments | | | | | | | |
| NW 1  MW mg/l:  NaHCO_3_ - 25.2; MgSO_4_ * 7H_2_O - 36.6;  CaCl_2_ * 6H_2_O - 233.8;  MgCO_3_ - 3.2  + 10% NW1 | 100 mg/l of bentonite clay (МWСl);  50 mg/l FeCl_3_ (МWI) | 1 g/L of sodium acetate and 1 g/l of glucose  for MWO and NWO series | Air  In  hermetically sealed vials | 30 | step-by-step filtration with syringe- filters 2.4, 1.2, 0.8, 0.4, 0.22, 0.1, and 0.05 µm –in diameter. | Elements in filtrate solution (including U): ICP-MS  Polysaccharides: phenol–sulfuric acid method  Protein: Folin phenol reagent according to Lowry  Total organic carbon: CHN analyzer  The size of the cells, colloidal particles, and zeta potential: the dynamic light scattering (Compact-Z particle size and zeta potential analyzer)  Eh, pH:  This data was used for PREEQC simulation |  |
| Model water experiments with actinides | | | | | | | |
| NW 1  MW  mg/l NaHCO_3_ - 25.2; MgSO_4_ * 7H_2_O - 36.6; CaCl_2_ * 6H_2_O - 233.8; MgCO_3_ - 3.2  + 10% NW1 | 100 mg/l of bentonite clay (МWСl)  50 mg/l FeCl_3_ (МWI)  10^-8^ M/l  233U,  237Np,  239Pu | 1 g/L of sodium acetate and 1 g/l of glucose  for MWO and NWO series | Air  In  hermetically sealed vials | 30 | step-by-step filtration with syringe- filters 2.4, 1.2, 0.8, 0.4, 0.22, 0.1, and 0.05 µm –in diameter | ^233^U, in filtrate solution: liquid scintillation,  ^239^Pu determination by alpha-spectrometry  ^237^Np concentration was determined by the luminescent method |  |

Table SI-1 Laboratory simulation tests description.

Table S2. Zeta potentials (mV) in natural and model water after addition of glucose.

| Sample | Time, days | | | |
| --- | --- | --- | --- | --- |
|  | 0 | 10 | 20 | 30 |
| MWO | -25.9 | -3.8 | -8.2 | -14.4 |
| NWO | -18.3 | 4.33 | -0.493 | -11.7 |

Table S3. The pH and Eh values during incubation on the model solutions

| Sample | pH | | | Eh, mV | | |
| --- | --- | --- | --- | --- | --- | --- |
|  | 0 days | 7 days | 21 day | 0 days | 7 days | 21 day |
| МV | 7.86 | 7.92 | 7.89 | 125 | 103 | 105 |
| МVО | 7.75 | 8.34 | 8.32 | 106 | -30 | -102 |
| МVСl | 8.03 | 8.08 | 8.11 | 75 | 35 | 31 |
| МVСlО | 7.86 | 8.86 | 8.46 | 76 | -22 | -83 |
| МVI | 8.1 | 8.15 | 8.21 | 25 | 2 | 13 |
| МVIO | 7.94 | 8.54 | 8.3 | 109 | -20 | -128 |
| NV | 8.88 | 8.62 | 8.7 | 141 | 39 | 0 |
| NVO | 8.65 | 8.68 | 8.58 | 146 | -43 | -136 |

Supplementary material

Table S1 Laboratory simulation tests description.

Table S2. Zeta potentials (mV) in natural and model water after addition of glucose.

Table S3. The pH and Eh values during incubation, model solutions.
